# Combined whole-body dynamic and static PET/CT with low-dose [^18^F]PSMA-1007 in prostate cancer patients

**DOI:** 10.1007/s00259-024-06620-1

**Published:** 2024-01-30

**Authors:** Christos Sachpekidis, Leyun Pan, Martin Groezinger, Dimitrios Stefanos Strauss, Antonia Dimitrakopoulou-Strauss

**Affiliations:** 1 Clinical Cooperation Unit Nuclear Medicine, German Cancer Research Center (DKFZ), Im Neuenheimer Feld 280, D-69210 Heidelberg, Germany; 2 Division of Radiology, German Cancer Research Center (DKFZ), Heidelberg, Germany; 3 Department of Diagnostic and Interventional Radiology (DIR), Heidelberg University Hospital, Heidelberg, Germany

**Keywords:** Prostate cancer, LAFOV PET/CT, Whole-body PET/CT, Dynamic PET, [^18^F]PSMA-1007, Kinetic modeling

## Abstract

**Aim:**

In addition to significant improvements in sensitivity and image quality, the recent introduction of long axial field-of-view (LAFOV) PET/CT scanners has enabled dynamic whole-body imaging for the first time. We aim herein to determine an appropriate acquisition time range for static low-dose [^18^F]PSMA-1007 PET imaging and to investigate the whole-body pharmacokinetics of [^18^F]PSMA-1007 by dynamic PET with the LAFOV Biograph Vision Quadra PET/CT in a group of prostate cancer patients.

**Methodology:**

In total, 38 prostate cancer patients were enrolled in the analysis for staging or re-staging purposes. Thirty-four patients underwent dynamic whole-body PET/CT (60 min) followed by static whole-body PET/CT and four patients underwent static whole-body PET/CT only. The activity applied was 2 MBq/kg [^18^F]PSMA-1007. The static PET images of 10-min duration (PET-10) were reconstructed and further split into 8-min (PET-8), 6-min (PET-6), 5-min (PET-5), 4-min (PET-4), and 2-min (PET-2) duration groups. Comparisons were made between the different reconstructed scan times in terms of lesion detection rate and image quality based on SUV calculations of tumor lesions and the spleen, which served as background. Analysis of the dynamic PET/CT data was based on a two-tissue compartment model using an image-derived input function obtained from the descending aorta.

**Results:**

Analysis of lesion detection rate showed no significant differences when reducing PET acquisitions from 10 up to 5 min. In particular, a total of 169 lesions were counted with PET-10, and the corresponding lesion detection rates (95% CI for the 90% quantile of the differences in tumor lesions) for shorter acquisitions were 100% (169/169) for PET-8 (95% CI: 0–0), 98.8% (167/169) for PET-6 (95% CI: 0–1), 95.9% (162/169) for PET-5 (95% CI: 0–3), 91.7% (155/169) for PET-4 (95% CI: 1–2), and 85.2% (144/169) for PET-2 (95% CI: 1–6). With the exception of PET-2, the differences observed between PET-10 and the other shorter acquisition protocols would have no impact on any patient in terms of clinical management. Objective evaluation of PET/CT image quality showed no significant decrease in tumor-to-background ratio (TBR) with shorter acquisition times, despite a gradual decrease in signal-to-noise ratio (SNR) in the spleen. Whole-body quantitative [^18^F]PSMA-1007 pharmacokinetic analysis acquired with full dynamic PET scanning was feasible in all patients. Two-tissue compartment modeling revealed significantly higher values for the parameter *k*_3_ in tumor lesions and parotid gland compared to liver and spleen, reflecting a higher specific tracer binding to the PSMA molecule and internalization rate in these tissues, a finding also supported by the respective time-activity curves. Furthermore, correlation analysis demonstrated a significantly strong positive correlation (*r* = 0.72) between SUV and *k*_3_ in tumor lesions.

**Conclusions:**

In prostate cancer, low-dose (2 MBq/kg) [^18^F]PSMA-1007 LAFOV PET/CT can reduce static scan time by 50% without significantly compromising lesion detection rate and objective image quality. In addition, dynamic PET can elucidate molecular pathways related to the physiology of [^18^F]PSMA-1007 in both tumor lesions and normal organs at the whole-body level. These findings unfold many of the potentials of the new LAFOV PET/CT technology in the field of PSMA-based diagnosis and theranostics of prostate cancer.

**Supplementary Information:**

The online version contains supplementary material available at 10.1007/s00259-024-06620-1.

## Introduction

PET/CT imaging with prostate-specific membrane antigen (PSMA)-targeted radioligands has emerged as an important diagnostic tool in prostate cancer (PC), outperforming conventional imaging modalities and previous generation radiopharmaceuticals in both primary staging and biochemical recurrence of the disease [1–6].

PSMA PET/CT imaging is routinely performed using a static protocol in which patient images are acquired at one time point, typically 60 min after tracer injection, although modified protocols with scans at later time points may improve image quality [7, 8]. Dynamic PET, on the other hand, allows continuous registration of pharmacokinetic information over time and, subsequently, accurate quantification of tracer uptake. However, its use in clinical practice is restricted by the long and complex acquisition protocols it entails. In addition, most dynamic PSMA PET studies have been performed using standard field of view (SAFOV) cameras, which limit dynamic sequences to one or two bed positions—usually over the pelvis—do not include large vessels within the FOV and do not allow whole-body pharmacokinetic measurements [9–13].

The recent introduction of long axial field of view (LAFOV) PET/CT systems has led to a substantial improvement in sensitivity and image quality, allowing for a significant reduction in acquisition time and/or low-dose examination protocols [14–21]. In dynamic PET, in particular, the new scanners dramatically enhance its capabilities, enabling for the first time the dynamic acquisition of the body trunk in a single measurement. This allows the simultaneous evaluation of radiotracer kinetics of most organs and tumor lesions, using large vessels for image-derived input function (IDIF) calculation, thus providing robust information on in vivo tracer biology [22, 23]. For PSMA PET imaging, this information may help to elucidate specific molecular pathways, such as the affinity between the PSMA radioligands and the receptor and the internalization of the radiotracers.

In the present study, we performed combined whole-body dynamic and static PET/CT scanning with the new LAFOV Biograph Vision Quadra PET/CT after application of low [^18^F]PSMA-1007 activity in PC patients with two main objectives: first, to determine an appropriate acquisition time range for low-dose static PC imaging by analyzing different emulated scan times and the quality of the resulting PET images, and, second, to assess the dynamic PET-derived whole-body pharmacokinetics of the tracer.

## Materials and methods

### Patients

A total of 38 consecutive PC patients (mean age 71.9 years, range 56–91 years) were enrolled in this retrospective analysis and underwent [^18^F]PSMA-1007 PET/CT for staging or re-staging purposes. In particular, 12 patients suffered from previously untreated PC, 22 patients showed biochemical PC recurrence after therapy with curative intent, and four patients showed biochemical disease progression under systemic treatment, as evidenced by PSA increase. In total, five patients had a history of previous androgen deprivation therapy (ADT). Patient characteristics are summarized in Table 1. The study was conducted in accordance with the Declaration of Helsinki and was approved by the Ethics Committee of the University of Heidelberg (S-253/2019). All patients gave written informed consent to undergo [^18^F]PSMA-1007 PET/CT according to the regulations of the German Medicinal Products Act §13(2b) and to have their medical records released.

**Table 1:** Characteristics of the patients investigated

Patient no	Age (years)	PSA (ng/mL)	Gleason score	Indication	Previous treatment	ADT at time of PET	Activity (MBq)	PET/CT protocol
1	67	0.9	7	Biochemical recurrence	Radical prostatectomy, lymphadenectomy, radiotherapy	No	136	Dynamic and static
2	71	2.3	7	Biochemical recurrence	Radical prostatectomy, lymphadenectomy	No	153	Dynamic and static
3	69	10.7	6	Primary staging	-	No	163	Dynamic and static
4	76	0.2	7	Biochemical recurrence	Radical prostatectomy, lymphadenectomy	No	160	Static
5	78	0.1	9	Biochemical recurrence	Radical prostatectomy, lymphadenectomy, radiotherapy	No	199	Dynamic and static
6	70	20.9	8	Biochemical recurrence	Radical prostatectomy, lymphadenectomy, radiotherapy	No	192	Dynamic and static
7	72	0.2	9	Biochemical recurrence	Radical prostatectomy, lymphadenectomy, radiotherapy	No	139	Dynamic and static
8	72	0.3	7	Biochemical recurrence	Radical prostatectomy	No	211	Dynamic and static
9	62	4.1	7	Primary staging	-	No	127	Dynamic and static
10	74	0.2	9	Biochemical recurrence	Radical prostatectomy, lymphadenectomy, radiotherapy	No	205	Dynamic and static
11	70	7.0	7	Biochemical recurrence	Radical prostatectomy	No	142	Dynamic and static
12	68	1.4	7	Biochemical recurrence	Radical prostatectomy	No	119	Dynamic and static
13	56	0.7	7	Biochemical recurrence	Radical prostatectomy, lymphadenectomy, brachytherapy	No	157	Static
14	61	12.5	6	Primary staging	-	No	165	Dynamic and static
15	81	4.9	7	Biochemical recurrence	Radiotherapy, ADT	Yes	228	Dynamic and static
16	78	0.3	7	Biochemical recurrence	Radical prostatectomy	No	180	Dynamic and static
17	91	6.6	7	Biochemical progression	ADT	Yes	179	Dynamic and static
18	65	4.3	7	Primary staging	-	No	185	Dynamic and static
19	84	25.1	7	Biochemical recurrence	Radiotherapy	No	182	Static
20	72	0.2	8	Biochemical recurrence	Radical prostatectomy, lymphadenectomy, radiotherapy	No	160	Dynamic and static
21	65	10.4	6	Primary staging	-	No	140	Dynamic and static
22	72	22.8	8	Primary staging	-	No	187	Dynamic and static
23	82	6.7	7	Primary staging	-	No	151	Dynamic and static
24	70	12.4	7	Primary staging	-	No	165	Dynamic and static
25	68	38.5	8	Biochemical recurrence	Radical prostatectomy	No	161	Dynamic and static
26	77	2.8	7	Biochemical recurrence	Radical prostatectomy	No	190	Dynamic and static
27	62	10.2	8	Primary staging	-	No	128	Dynamic and static
28	67	0.2	9	Biochemical recurrence	Radical prostatectomy	No	149	Dynamic and static
29	75	9.8	6	Primary staging	-	No	157	Dynamic and static
30	72	0.5	9	Biochemical recurrence	Radical prostatectomy, lymphadenectomy	No	167	Dynamic and static
31	58	44.0	9	Primary staging	-	No	165	Dynamic and static
32	71	13.0	9	Primary staging	-	No	224	Dynamic and static
33	73	0.2	7	Biochemical progression	Radical prostatectomy, radiotherapy, ADT	Yes	151	Dynamic and static
34	79	4.8	7	Biochemical progression	Radical prostatectomy, radiotherapy, ADT	Yes	162	Dynamic and static
35	86	3.7	7	Biochemical recurrence	Radiotherapy	No	163	Static
36	72	0.9	7	Biochemical recurrence	Radical prostatectomy, radiotherapy	No	193	Dynamic and static
37	64	0.4	7	Biochemical recurrence	Radical prostatectomy	No	180	Dynamic and static
38	83	0.3	9	Biochemical progression	Radical prostatectomy, ADT	Yes	165	Dynamic and static

*ADT*, androgen deprivation therapy

### PET/CT examination

Patients underwent PET/CT with a LAFOV scanner (Biograph Vision Quadra, Siemens Co., Erlangen, Germany) after intravenous administration of a body weight adjusted activity of 2 MBq/kg [^18^F]PSMA-1007 (median 165 MBq; range 119–228 MBq).

PET/CT data acquisition consisted of the dynamic part, performed in 34 patients of the cohort, and the static part, performed in all 38 patients. Dynamic PET/CT was performed from the top of the head to the upper thigh (FOV 106 cm) for 60 min after i.v. injection of the radiotracer using a 33-frame protocol (10 frames of 15 s, 5 frames of 30 s, 5 frames of 60 s, 5 frames of 120 s, and 8 frames of 300 s).

After completion of the dynamic PET acquisition, the patients were asked to urinate and then additional total-body imaging (starting at 70 min after tracer injection) from the skull through the feet was performed in two bed positions (each FOV 106 cm): the first bed position covered the area from the top of the head to the upper thigh (10-min acquisition in list mode; PET-10), and the second bed position covered the lower extremities (5-min acquisition in list mode). The PET images of the first bed position (head to upper thigh) were first reconstructed using the entire 10-min data and were further subdivided into 8-min (PET-8), 6-min (PET-6), 5-min (PET-5), 4-min (PET-4), and 2-min (PET-2) duration groups to compare different acquisition times for fast acquisition scenarios. All PET images were attenuation corrected and an image matrix of 440 × 440 pixels was used for iterative image reconstruction. Images were reconstructed using the manufacturer’s standard reconstruction method (Siemens Healthineers) using the point spread function + time-of-flight algorithm (PSF + TOF, 4 iterations × 5 subsets) without Gaussian filtering into 1.65 × 1.65 × 1.65 mm^3^ voxels.

A low-dose attenuation CT (120 kV, 30 eff. mA) was used for attenuation correction of the dynamic emission PET data and for image fusion. A second low-dose CT (120 kV, eff. 30 mA) was performed after completion of the dynamic series covering the area from the skull to the feet in order to counteract patient movement after dynamic PET.

### Data analysis

#### Visual assessment of static PET/CT scans

Static image analysis was performed using a dedicated imaging workstation and software (aycan Osirix^PRO^). Two experienced, board-certified nuclear medicine physicians well versed in PC diagnosis with PSMA radioligands (CS, ADS) read the datasets together and any disagreements were resolved by consensus.

Visual analysis was based on the identification of sites of focally enhanced [^18^F]PSMA-1007 uptake relative to local background, which were considered suggestive of PC involvement (tumor lesions) after disregarding known benign [^18^F]PSMA-1007 avid structures, such as ganglia, ureters, and sites of unspecific bone uptake. The number of tumor lesions was determined in each scan, with a maximum of up to 20 lesions measured per patient. With regard to lesion detectability, the results of the 10-min PET acquisition (PET-10) served as a reference against which the results of the other duration groups (PET-8, PET-6, PET-5, PET-4, PET-2) were compared.

#### Objective evaluation of static PET/CT image quality

Objective assessment of PET/CT image quality was based on volumes of interest (VOIs) and subsequent calculation of SUV values (SUV_mean_, SUV_max_) in tumor lesions and the background using a dedicated software (PMOD Technologies, Zurich, Switzerland) (http://www.pmod.com/files/download/v31/doc/pbas/4729.htm). In specific, SUV evaluation of tumor lesions was based on VOIs drawn with an isocontour mode (pseudo-snake) over sites of focally enhanced [^18^F]PSMA-1007 uptake suggestive of PC. Due to the liver-dominant excretion of the radioligand, background measurements were made in the spleen after drawing VOIs over the organ, also using an isocontour mode [24]. VOIs were copied and pasted between different images obtained from different (list mode) frame durations, ensuring that the same VOI was analyzed for each acquisition as previously described [25]. The tumor-to-background ratio (TBR) was defined as the SUV_mean_ of the tumor lesion divided by the SUV_mean_ of the spleen background. Moreover, the signal-to-noise ratio (SNR) of the background (spleen) was measured as the SUV_mean_ of the background divided by its standard deviation (SD).

It should be noted that the comparison of the results, both of visual analysis and objective evaluation of PET/CT image quality, was focused on the first bed position, which covered the area from the top of the head to the upper thigh.

#### Evaluation of dynamic PET/CT data

Evaluation of the dynamic PET/CT data was also based on VOIs drawn over tumor lesions and normal organs [25–27]. In particular, tumor lesions were assessed using irregular VOIs drawn using an isocontour mode and placed over the entire lesions, which were further classified into prostate lesions, lymph node metastases, bone metastases, and soft tissue metastases. For normal organs, the parotid gland and the spleen were assessed after drawing VOIs over the entire organ using an isocontour mode, while the liver was assessed after placing spherical VOIs covering approximately five consecutive slices over the right liver lobe. Blood pool calculations were obtained from the average of the descending aorta VOI data, consisting of at least seven slices in sequential PET/CT images, placed centrally in the lumen of the aorta without including the aortic wall.

Semi-quantitative evaluations were performed based on SUV calculations 50–60 min after tracer injection (the average SUV of the last two frames of the dynamic PET acquisition) generated from the VOIs placed over tumor lesions and normal organs. In addition, a detailed quantitative evaluation of the pharmacokinetics of [^18^F]PSMA-1007 derived from the entire 60-min dynamic PET acquisition in tumor lesions and normal organs with high tracer uptake, including the spleen, liver, and parotid gland, was performed using a reversible two-tissue compartment model. The IDIF used for whole-body kinetic modeling was obtained from the descending aorta VOI data. This compartment model includes the plasma compartment (c_plasma_), the free (unbound) component of [^18^F]PSMA-1007 in the interstitial and/or intracellular space (c_1_), and the PSMA-specific component of the radiotracer (c_2_) (9). The application of two-tissue compartment modeling leads to the extraction of the parameters *K*_1_ (mL/ccm/min), *k*_2_ (min^−1^), *k*_3_ (min^−1^), and *k*_4_ (min^−1^). In particular, *K*_1_ and *k*_2_ reflect the forward and reverse transports of the radiotracer between plasma and the “reversible” interstitial/intracellular compartment, *k*_3_ is associated with tracer binding to PSMA and its internalization via clathrin-mediated endocytosis, and *k*_4_ represents the dissociation of the tracer from PSMA and its externalization. Furthermore, the global tracer influx *K*_i_ (mL/ccm/min) was calculated from the compartment data using the formula *K*_i_ = (*K*_1_ × *k*_3_)/(*k*_2_ + *k*_3_). The application of whole-body dynamic PET/CT scanning also led to the extraction of time-activity curves (TACs) from tumor lesions and normal organs, showing the activity concentration of [^18^F]PSMA-1007 in the selected VOIs during the 60 min of dynamic PET/CT acquisition.

Besides compartmental modeling, fractal analysis, a non-compartmental model, was used to calculate the parameter of heterogeneity and complexity, expressed as a non-integer value called fractal dimension (FD). FD computation is performed in each individual voxel of a VOI and is based on the box-counting procedure of chaos theory. The values of FD vary from 0 to 2, indicating the more deterministic or chaotic distribution of tracer activity over time [28].

### Statistical analysis

Continuous variables were expressed as mean ± standard deviation (SD). For visual (qualitative) assessment of static PET/CT scans in terms of lesion detection rate, the 95% confidence intervals (95% CI) for the 90% quantile of the differences in the number of tumor lesions between the different PET acquisition times and the PET-10 reference were calculated using the quantileCI function from the R package MKinfer. Further, differences between parameters employed for objective evaluation of static PET/CT image quality as well as between kinetic parameters of tumor lesions and normal organs derived from dynamic PET data were evaluated using the paired Student’s *t*-test. Correlations between kinetic parameters and SUV were investigated using Spearman’s rank correlation analysis. Statistical significance was considered for *p*-values less than 0.05. Statistical analysis was performed in R (version 4.0.3) andStata/MP 14.2 (StataCorp LLC).

## Results

### Visual assessment of static PET/CT and comparison between different static acquisition protocols

Based on the results of PET-10, a total of 35/38 positive (92.1%) and three negative (7.9%) scans were diagnosed. The respective numbers of positive PET/CT scans (at least one [^18^F]PSMA-1007-avid tumor) in the shorter acquisitions were the same as for PET-10, i.e., 35/38 (92.1%) for the PET-8, PET-6, PET-5, and PET-4 acquisitions, whereas in the PET-2 acquisitions 34/38 (89.5%) scans were positive.

**Fig. 1:**
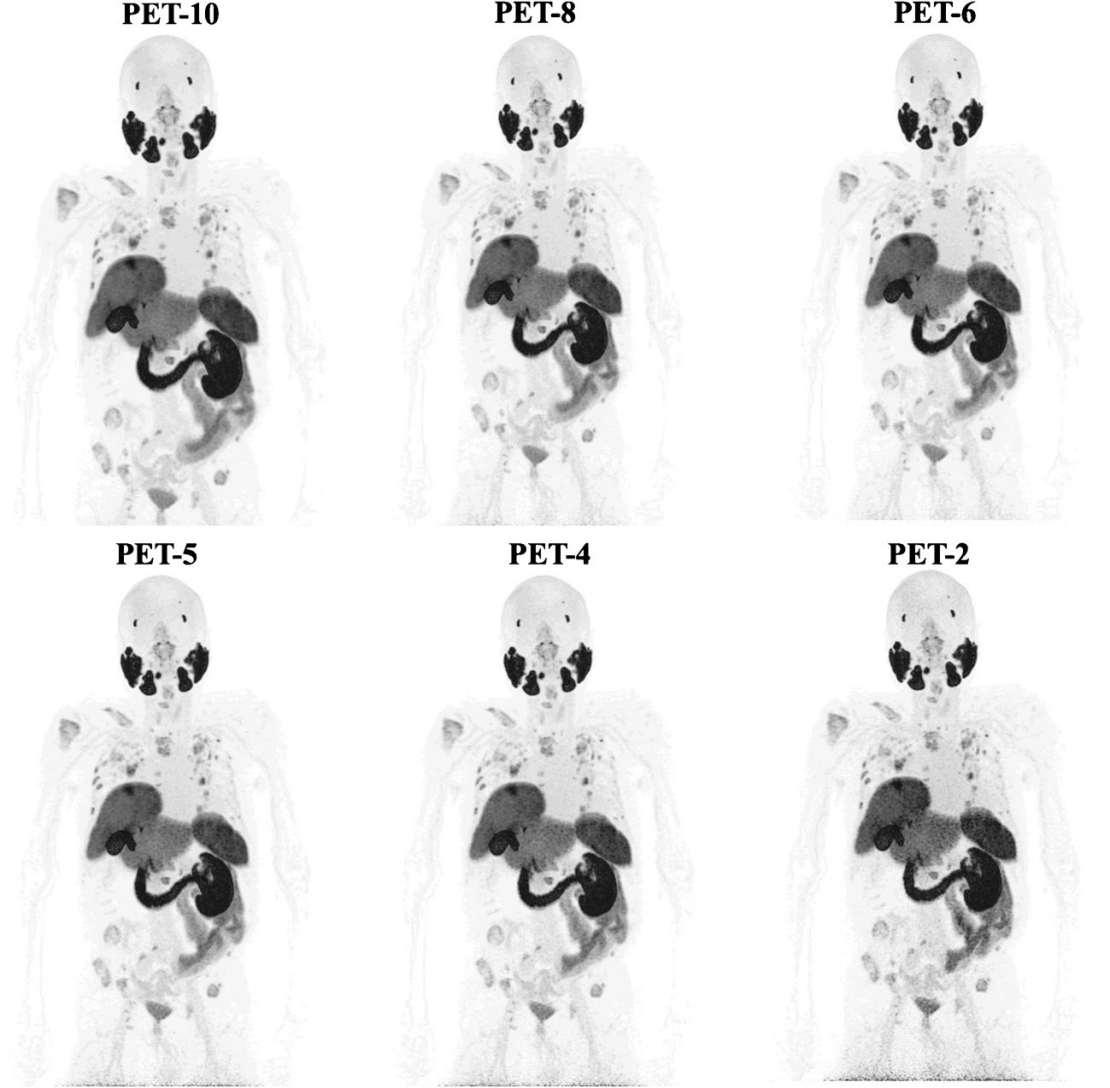
Maximum intensity projection (MIP) [^18^F]PSMA-1007 PET images of a 70-year-old patient referred for imaging due to biochemical recurrence (PSA 20.9 ng/mL) of PC after radical prostatectomy and radiotherapy. Presented are the PET-10, PET-8, PET-6, PET-5, PET-4, and PET-2 acquisitions. All acquisitions clearly demonstrate disseminated [^18^F]PSMA-1007 avid metastastic disease with multiple lymph, node, bone, and soft tissue metastases. Of note is the almost complete lack of tracer uptake in the right kidney due to polycystic kidney disease and the hypertrophic left lobe of the liver

Regarding the lesion detection rate, extensive metastatic involvement with > 20 [^18^F]PSMA-1007-avid tumor lesions was observed in four patients in all PET acquisitions, making the exact calculation very difficult. In the remaining 34 patients for whom the exact calculation of [^18^F]PSMA-1007-avid tumor lesions was feasible, a total of 169 lesions were detected in the PET-10 scans. Compared to the reference of PET-10 images, the lesion detection rates of the PET-8, PET-6, PET-5, PET-4, and PET-2 images were 100% (169/169), 98.8% (167/169), 95.9% (162/169), 91.7% (155/169), and 85.2% (144/169), respectively. The 95% CI for the 90% quantile of the differences in the number of tumor lesions between the different PET acquisition times and the reference of PET-10 were as follows: 0–0 (PET-8), 0–1 (PET-6), 0–3 (PET-5), 1–4 (PET-4), and 1–6 (PET-2). Notably, with the exception of PET-2, the differences observed between PET-10 and the other shorter acquisition protocols would have no clinical or therapeutic consequences for any of the patients studied, as they would not lead to differences in staging or restaging of the disease. Table 2 shows the results of visual assessment of PET/CT scans in terms of lesion detection rate. Figure 1 presents an example of [^18^F]PSMA-1007 PET images from a patient assessed with different time acquisition protocols.

**Table 2:** Results of visual analysis of static PET/CT images in terms of lesion detection rate

Measurement	PET-10	PET-8	PET-6	PET-5	PET-4	PET-2
Lesion detection rate^a^	n.a	169/169 (100%)	167/169 (98.8%)	162/169 (95.9%)	155/169 (91.7%)	144/169 (85.2%)
95% CI for 90% quantile of differences of number of lesions^a^	n.a	0–0	0–1	0–3	1–4	1–6

After excluding the three scans in which the exact calculation of tumor lesions was very difficult due to the extent of metastatic involvement (> 20 lesions)

*n.a.*, not applicable

^a^Compared to the reference of PET-10

### Objective evaluation of static PET/CT image quality and comparison between different time acquisition protocols

**Fig. 2:**
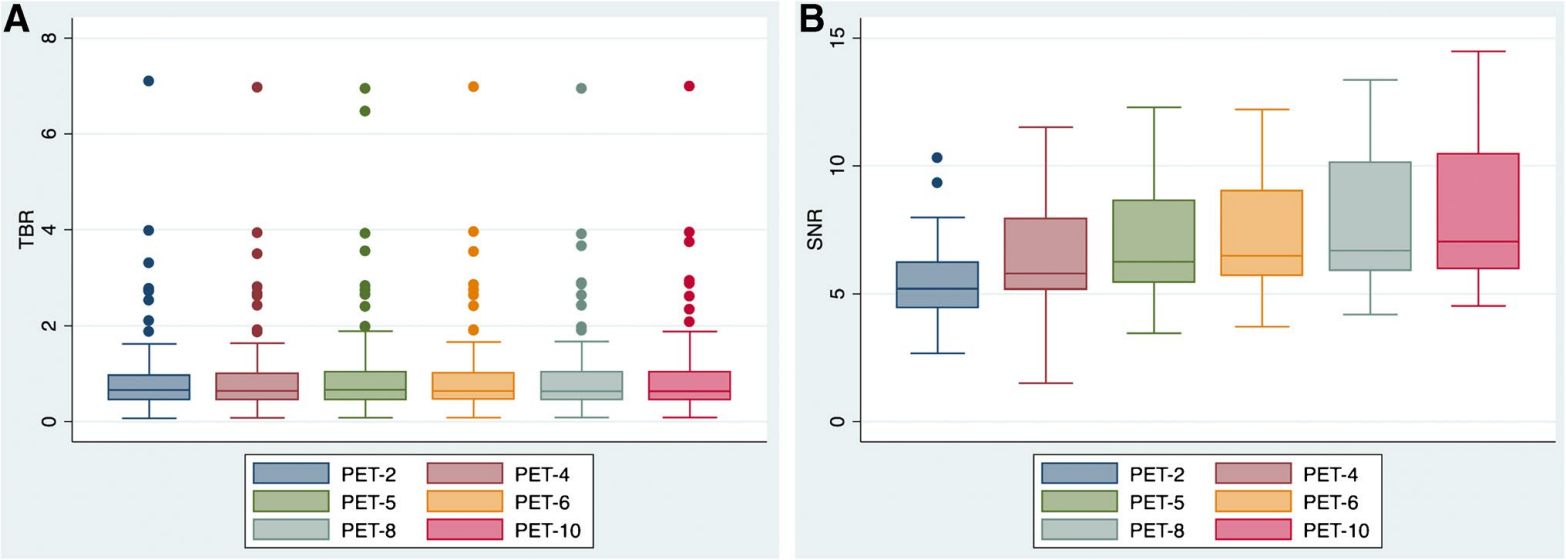
Box plots for comparison of objective image quality parameters among the different PET acquisition groups by means of TBR (**A**) and SNR (**B**). No differences in TBR are observed between the different acquisition times, whereas SNR significantly decreases while moving from longer to shorter-duration protocols

In static PET/CT, performed 70 min after tracer administration, a total of 133 tumor lesions were semi-quantitatively evaluated. The resulting SUV values (SUV_mean_, SUV_max_) showed only minimal differences between the different acquisition protocols, which was also reflected in the TBR calculations, showing no significant decrease in TBR with any of the shorter duration protocols compared to the 10-min protocol. Furthermore, as a general trend, spleen SNR decreased with decreasing acquisition time, with the SNR calculated in the PET-10 images being significantly higher than all other acquisitions. The results of the objective image quality assessment are presented in Fig. 2 and in Supplementary Table 1.

### Evaluation of dynamic PET/CT data

**Fig. 3:**
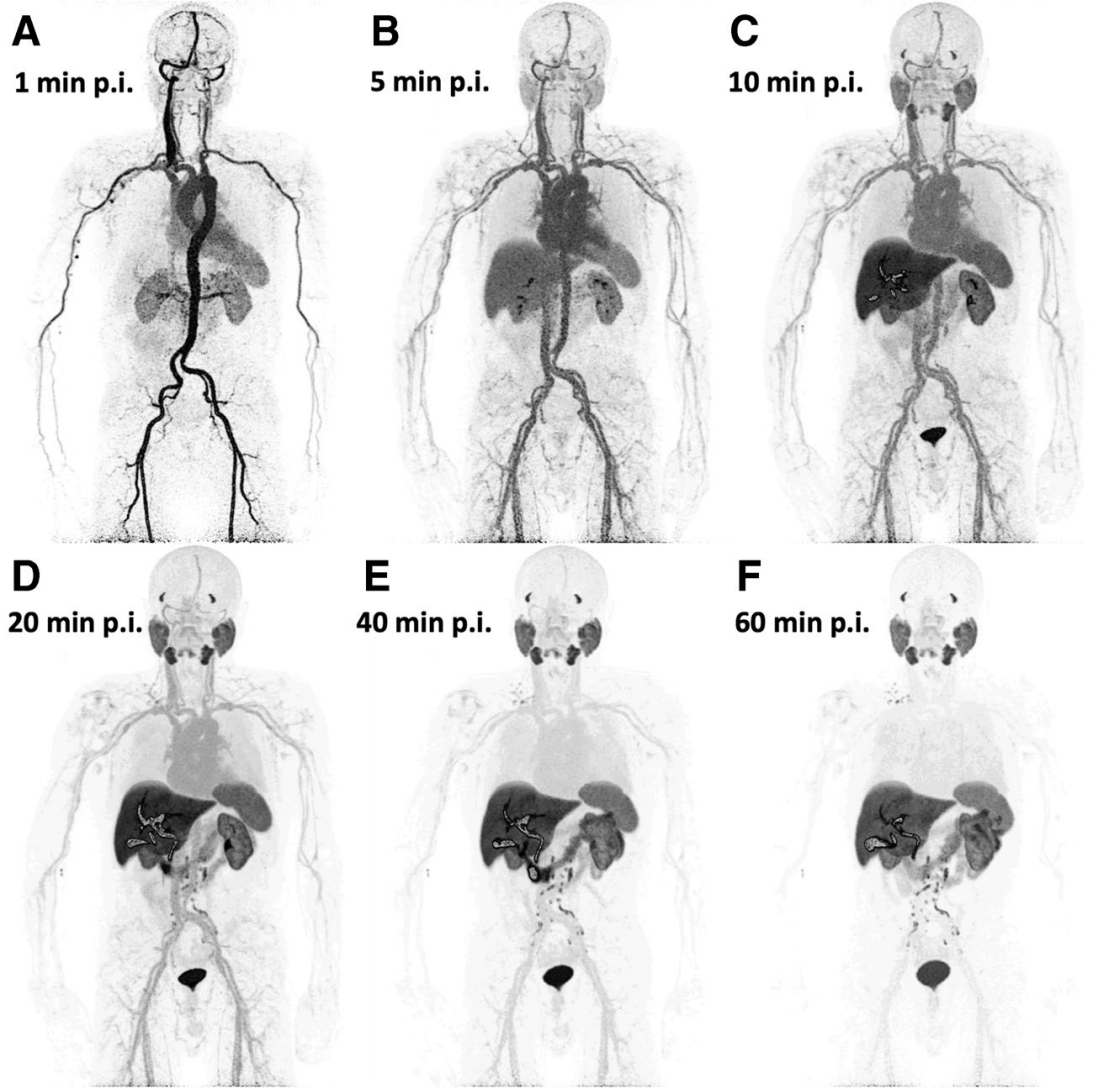
Example of whole-body dynamic images acquired at different time points of dynamic PET acquisition of a patient with biochemical recurrence of PC. The images shown depict acquisitions at 1 (**A**), 5 (**B**), 10 (**C**), 20 (**D**), 40 (**E**), and 60 min (**F**) after administration of the radiopharmaceutical (p.i., post-injection). The patient has multiple iliac, retroperitoneal, and supraclavicular [^18^F]PSMA-1007 avid lymph node metastases. Notably, some retroperitoneal lymph node metastases can be delineated already 10 min after tracer injection

No further lesions were detected on whole-body dynamic PET/CT images compared to static PET/CT. One hundred and five (105) [^18^F]PSMA-1007-positive tumor lesions, including 23 prostate lesions (primary and locally recurrent PC lesions), 30 lymph node metastases, 49 bone metastases, and three soft tissue metastases, as well as normal organs, including the parotid gland, liver, and spleen, were evaluated both semi-quantitatively and quantitatively by means of dynamic PET/CT. The results of the semi-quantitative and quantitative evaluation are presented in Tables 3 and 4. An example of whole-body dynamic images acquired at different time points of dynamic PET acquisition of a patient with biochemical recurrence of PC is provided (Fig. 3).

**Table 3:** Descriptive statistics of dynamic PET data of [^18^F]PSMA-1007 in normal organs and tumor lesions. SUV refers to the average uptake value calculated from the dynamic acquisitions performed 50–60 min after injection. The kinetic parameters were calculated from the entire 60-min dynamic acquisition. *P* values refer to the comparison of the semi-quantitative and quantitative parameters of normal organs with tumor lesions

	SUV Mean ± SD	*K*_1_ (mL/ccm/min) Mean ± SD	*k*_2_ (min^−1^) Mean ± SD	*k*_3_ (min^−1^) Mean ± SD	*k*_4_ (min^−1^) Mean ± SD	Influx, *K*_i_ (mL/ccm/min) Mean ± SD	FD Mean ± SD
Spleen	8.34 ± 2.56	0.746 ± 0.717*	2.533 ± 1.882*	0.178 ± 0.201**	0.003 ± 0.003	0.030 ± 0.011	1.321 ± 0.1*
Liver	10.24 ± 2.86*	0.490 ± 0.3*	1.344 ± 0.905*	0.118 ± 0.031**	0.011 ± 0.006	0.039 ± 0.01*	1.373 ± 0.023*
Parotid gland	14.56 ± 4.54*	0.178 ± 0.16*	1.426 ± 1.663*	0.524 ± 0.27	0.031 ± 0.171	0.046 ± 0.02*	1.380 ± 0.077*
Tumor lesions	7.56 ± 5.88	0.136 ± 0.14	0.924 ± 0.876	0.221 ± 0.199	0.036 ± 0.498	0.023 ± 0.023	1.274 ± 0.108

*FD*, fractal dimension

*Significantly higher value for normal organs than tumor lesions (*p* < 0.05)

**Significantly higher value for tumor lesions than normal organs (*p* < 0.05)

**Table 4:** Descriptive statistics of dynamic PET data of [^18^F]PSMA-1007 in different classes of tumor lesions. SUV refers to the average uptake value calculated from the dynamic acquisitions performed 50–60 min after injection. The kinetic parameters were calculated from the entire 60-min dynamic acquisition

	SUV Mean ± SD	*K*_1_ (mL/ccm/min) Mean ± SD	*k*_2_ (min^−1^) Mean ± SD	*k*_3_ (min^−1^) Mean ± SD	*k*_4_ (min^−1^) Mean ± SD	Influx, *K*_i_ (mL/ccm/min) Mean ± SD	FD Mean ± SD
Prostate lesions	8.22 ± 4.39^#^	0.105 ± 0.059	0.581 ± 0.296	0.2 ± 0.13^#^	0.002 ± 0.004	0.024 ± 0.014^#^	1.281 ± 0.135^#^
Lymph node metastases	12.72 ± 7.52*	0.186 ± 0.199	1.319 ± 1.349	0.42 ± 0.243*	0.17 ± 0.922	0.041 ± 0.034*	1.357 ± 0.082*
Bone metastases	4.17 ± 1.73	0.125 ± 0.121	0.863 ± 0.593	0.107 ± 0.063	0.019 ± 0.114	0.012 ± 00.6	1.219 ± 0.071

Due to their small number (*n* = 3), soft tissue metastases were not included in the statistical analysis

*Significantly higher values for lymph node metastases compared to prostate lesions and bone metastases (*p* < 0.05)

^#^Significantly higher values for prostate lesions compared to bone metastases (*p* < 0.05)

In brief, the average SUV calculated from dynamic PET between 50 and 60 min after [^18^F]PSMA-1007 injection was significantly lower for tumor lesions compared to parotid gland and liver, with no significant differences between tumors and spleen. Compartment modeling leading to extraction of the corresponding kinetic metrics, revealed significantly lower *K*_1_ and *k*_2_ values for tumor lesions compared to all evaluated normal organs. For *k*_3_, tumor lesions showed significantly higher values than liver and spleen and no significant differences with parotid gland. As expected, *k*_4_ values were minimal across all tissues with no differences between them. Similar to SUV, tracer influx was significantly lower in tumor lesions than in parotid gland and liver, and comparable to spleen. Finally, FD was higher in normal organs than tumor lesions.

Among tumor lesions, lymph node metastases showed the highest SUV, *k*_3_, influx and FD values, followed by prostate lesions, which showed significantly higher values than bone metastases (Table 4). No differences were observed between different classes of tumor lesions for the remaining kinetic parameters, *k*_2_ and *k*_4_.

Correlation analysis showed no significant correlation between *K*_1_, *k*_2_ and SUV for any tissue, normal or tumor. The parameter *k*_3_ showed a significant moderate positive correlation with SUV in the parotid gland (*r* = 0.57) and the liver (*r* = 0.51) but not in the spleen (*r* = 0.16), with the strongest correlation observed in tumor lesions (*r* = 0.72). Conversely, *k*_4_ showed a significant moderate negative correlation with SUV in the parotid gland (*r* =  − 0.40) and the liver (*r* =  − 0.42), and a weak negative correlation in tumor lesions (*r* =  − 0.26). Finally, tracer influx (*K*_i_) correlated significantly with SUV in all tissues, again with tumor lesions showing the strongest correlation of all tissues (*r* = 0.93) (Table 5).

**Table 5:** Results of the Spearman’s rank correlation analysis (correlation coefficient *r* values) between SUV and kinetic parameters of [^18^F]PSMA-1007 for each tissue. SUV refers to the average uptake value calculated from the dynamic acquisitions performed 50–60 min after injection. The kinetic parameters were calculated from the entire 60-min dynamic acquisition

	*K*_1_	*k*_2_	*k*_3_	*k*_4_	Influx, K_i_
Spleen SUV	0.16	0.06	0.16	− 0.16	0.76*
Liver SUV	0.22	0.12	0.51*	− 0.42*	0.76*
Parotid gland SUV	0.22	− 0.15	0.57*	− 0.40*	0.91*
Tumor lesions SUV	0.05	− 0.08	0.72*	− 0.26*	0.93*

*Statistically significant correlation for each tissue (*p* < 0.05)

**Fig. 4:**
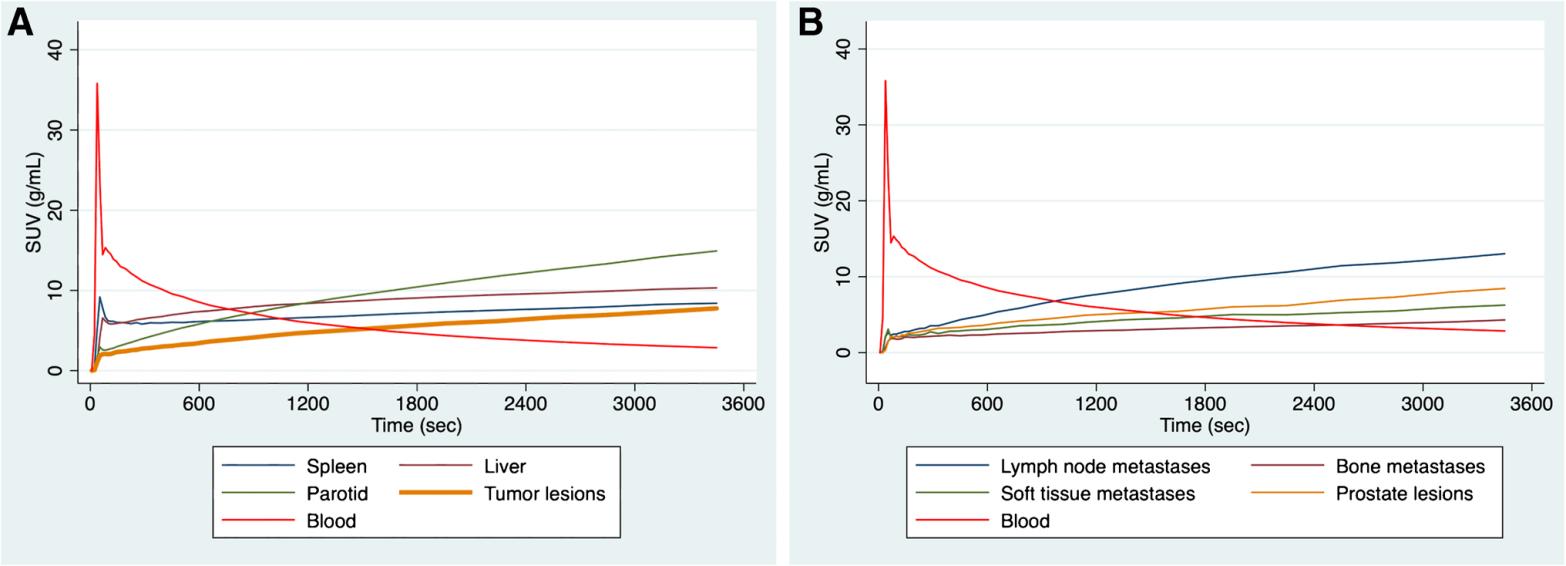
Time-activity curves (TACs) derived from whole-body dynamic PET/CT studies using a 33-frame protocol (*y*-axis, average SUV; *x*-axis, time in seconds). The TACs represent the mean values of all evaluated VOIs corresponding to normal organs and tumor lesions. **A** TACs of the blood pool (descending aorta), spleen, liver, parotid gland, and tumor lesions. **B** TACs of the blood pool and tumor lesions divided in different classes (prostate lesions, lymph node metastases, bone metastases, soft tissue metastases)

Dynamic PET/CT scanning also led to the generation of TACs depicting the activity concentration of [^18^F]PSMA-1007 during dynamic PET/CT acquisition (33 time points according to the defined frames). In general, the curves derived from both tumor lesions and normal organs showed an increasing radiotracer concentration over time. In particular, the spleen and liver showed a high initial peak of the tracer after the injection of the radiopharmaceutical, whereas tumor lesions and the parotid gland exhibited lower initial peaks but the highest curve slopes (Fig. 4A). With regard to tumor lesions, lymph node metastases showed the highest increase in tumor uptake over time, followed by prostate lesions. Bone metastases showed the lowest curve slope among tumor lesions (Fig. 4B).

## Discussion

Besides the marked improvement in sensitivity and image quality, the advent of long axial field-of-view (LAFOV) PET/CT scanners has enabled the performance of whole-body dynamic imaging, eliminating the need for multiple bed position protocols or continuous bed motion. This development renders for the first time feasible to capture tracer pharmacokinetics in the major organs and most tumor lesions with high temporal resolution. The present work describes our initial results from combined, whole-body dynamic, and static PET/CT scanning with the new LAFOV Biograph Vision Quadra PET/CT after application of low [^18^F]PSMA-1007 activity in PC patients.

The main findings of our analysis are as follows: firstly, reducing static PET acquisition times by 50% in low-dose [^18^F]PSMA-1007 LAFOV PET/CT is possible and can be performed safely both in terms of scan interpretation and objective image quality evaluation. Secondly, whole-body quantitative [^18^F]PSMA-1007 kinetics analysis acquired with full dynamic PET scanning is feasible, resulting in kinetic metrics which reflect molecular pathways related to the physiology of the radioligand. In particular, we were able to show that the specific binding to PSMA molecules and the internalization rate of the radiotracer is higher in tumor lesions and the parotid gland than in the spleen and liver.

A growing body of evidence in different tumors attests to the potential of achieving faster PET imaging and/or reducing the administered radiopharmaceutical activity, while maintaining a comparable image quality with the new LAFOV systems [19–21, 27, 29, 30]. Based on this experience, we, herein, aimed to determine an appropriate acquisition time range for static PET/CT imaging in a cohort of PC patients studied after application of 2 MBq/kg [^18^F]PSMA-1007, and not 3–4 MBq/kg as per EANM/SNMMI guidelines [31]. Our results confirm previous findings and show that the new scanner allows a significant reduction in acquisition time for static imaging by at least 50%, despite lower administered activity, without negatively impacting patient assessment. In particular, although more lesions were detected with 10-min PET acquisitions, i.e., longer scans are of utility, reducing the PET acquisition duration from 10 to 5 min had no significant adverse impact on the lesion detection rate. This is reflected by the rather stringent criterion of calculating the respective 95% CI for the 90% quantile of the differences between different acquisition protocols. In addition, the reduction in PET scan time to 4 min would not have had any potential impact on staging, restaging, and subsequent management in any patient, consistent with a recent study in head and neck cancer using standard [^18^F]FDG activity [32]. Based on the above, the combination of the specific applied activity (2 MBq/kg) with the specific scan duration (5 min) represents a clinically acceptable approach for imaging on a LAFOV system.

Apart from the visual assessment of PET image quality, we investigated objective image quality parameters, namely, TBR and SNR. Similar to previous results from our group [25], shortening PET acquisition times was associated with a decrease in SNR, whereas reducing the acquisition protocol to 2 min did not result in any significant differences in TBR, also in agreement with previous studies using LAFOV PET/CT [17, 18]. This potential to reduce the duration of static PET acquisition while maintaining image quality is one of the major advantages of the new LAFOV scanners, with benefits both for the patient, through improved comfort and reduced motion artifacts, and for the operation of nuclear medicine departments, in terms of increased patient throughput.

Despite their indisputable clinical applicability, static PET acquisitions and the associated semi-quantitative estimates of SUV are constrained by the limitations of subjective image interpretation and the influence of many factors on SUV measurements, respectively [33]. In this context, dynamic PET imaging and kinetic modelling can provide significantly more information about in vivo biology by delineating both the temporal and spatial patterns of tracer uptake, while mitigating several potential sources of error associated with static imaging [34]. Indeed, earlier research has highlighted the potential of dynamic PET to investigate specific molecular pathways related to the physiology of the PSMA radioligands such as receptor availability, radioligand-receptor affinity, binding, and internalization, as well as dynamics of tracer accumulation [9–13]. However, most studies to date have been performed with conventional SAFOV PET systems, limiting the dynamic protocol to one or two bed positions, usually over the pelvis. This approach has the disadvantages of missing tissues in other parts of the body and reducing the robustness of the IDIF calculation by excluding the heart and large vessels from the dynamic PET FO [21]. Notably, in a recent dynamic study with PSMA tracers and conventional SAFOV PET, dynamic imaging was performed over the chest region, an approach that leads to the generation of a robust IDIF but neglects the more clinically relevant for PC pelvic region [35].

These drawbacks can now be overcome with the employment of the LAFOV scanners. For the first time, we are able to use large vessels for IDIF calculations, and to study the pharmacokinetics of PSMA radioligands in all tumor lesions of the body trunk and in organs located outside the pelvis. In our study, kinetic analysis revealed significantly higher *K*_1_ values for the spleen, liver, and parotid gland compared to tumor lesions. This indicates a faster transport of [^18^F]PSMA-1007 between plasma and the “reversible” interstitial/intracellular compartment and a higher amount of free unbound tracer in normal organs than in PC, probably due to the tracer concentration in the blood pool, as also highlighted by the generated TACs. This finding is consistent with the results of a recent study on [^68^Ga]PSMA-11 PET/CT [36]. On the other hand, *k*_3_ values were significantly higher for tumor lesions compared to liver and spleen, reflecting a higher specific tracer binding to PSMA and internalization rate in PC than in these organs. This finding is supported by the strong correlation between *k*_3_ and SUV in tumor tissue, and proves the higher, more specific binding of [^18^F]PSMA-1007 to the PSMA molecule in PC cells compared to these reference organs.

Notably, the parameter *k*_3_ was significantly higher in the parotid gland than in the rest normal tissues and did not differ significantly from tumor lesions. This finding is of particular importance in PSMA-radioligand therapy (RLT), where salivary gland toxicity is a major challenge [37, 38]. This high internalization rate reflects the rather specific binding of the radioligand in the gland and partly explains the high frequency of salivary gland toxicity observed in RLT. In this setting, a potential application of whole-body dynamic PET could be the stratification of patient candidates for RLT based on the dynamic uptake pattern and the degree of binding and internalization of PSMA radiopharmaceuticals in both tumor lesions and the parotid gland.

To our knowledge, this is the first study on whole-body dynamic PET/CT with the tracer [^18^F]PSMA-1007 and a LAFOV scanner. Recently, the first results from the use of whole-body dynamic PSMA-radioligand PET imaging using the tracer [^68^Ga]-PSMA-11 have been published. Specifically, employing the total-body uEXPLORER scanner (United Imaging Healthcare) in small PC cohorts, the Shangai group studied the TACs and pharmacokinetics with the use of compartment modeling. In line with our results, they showed that the parameter *k*_3_ exhibited the highest performance in distinguishing between physiological and pathological [^68^Ga]Ga-PSMA-11 uptake, thus highlighting the potential of this approach in differential diagnosis issues related to PSMA imaging [23, 36].

We note some weaknesses of our study. Firstly, this is a single-center retrospective analysis of a relatively small patient cohort, including few patients (*n* = 5) on ADT, which influences PSMA expression [39–41]. Thus, a validation of the herein presented findings in the context of a larger, multicenter, prospective trial would be required. A second limitation is the lack of histological confirmation of the vast majority of the [^18^F]-PSMA-1007 avid focal lesions, with the exception of biopsy-proven primary tumors. Clearly, the use of histopathological findings as a reference is a more reliable method than referring to static PET-10 images; however, this is obviously not possible in the clinical setting. Moreover, previous studies have demonstrated high correlation between imaging and histopathologic findings for PSMA tracers [42, 43] with the exception of some non-specific [^18^F]-PSMA-1007 avid bone lesions detected rather frequently with digital PET scanners [44, 45], which were interpreted with caution in our analysis. Thirdly, lesion detection in the different acquisition protocols of static PET/CT images was based on a consensus read between two physicians. A multi-reader assessment of the scans would probably have provided more robust results. To reduce recall bias, reading of PET/CT scans for different acquisition protocols for the same examination was performed at least one week apart. Finally, in the present study the evaluation of the dynamic PET data was mainly based on two-tissue compartment modeling and did not include parametric imaging, which would visualize specific features of the radiotracer kinetics. However, this will be the subject of a future study of our group.

## Conclusion

We performed combined dynamic and static whole-body PET/CT scans with the new LAFOV Biograph Vision Quadra PET/CT in PC patients after administration of 2 MBq/kg [^18^F]PSMA-1007. Our results demonstrate that at these lower radiotracer activities, a 50% reduction in PET acquisition time with LAFOV PET/CT can meet clinical requirements without compromising lesion detection rates for static imaging. In addition, whole-body quantitative [^18^F]PSMA-1007 kinetics analysis acquired with full dynamic PET scanning is feasible, with compartmental modeling highlighting higher specific binding to the PSMA receptor and faster internalization of the radioligand in tumor lesions and the parotid gland than in the spleen and liver. The presented findings unfold many of the potentials of the new LAFOV PET/CT technology, both in terms of improving the quality and speed of image acquisition, even at lower applied activities, and by providing, for the first time, whole-body [^18^F]PSMA-1007 pharmacokinetic data, with potential clinical implications in the field of PC diagnosis and theranostics.

### Supplementary Information

Below is the link to the electronic supplementary material.

Supplementary file1 (DOCX 14 KB)

## Data Availability

The datasets generated during and/or analyzed during the current study are available from the corresponding author on reasonable request.
