# Supplementary material for: Combined whole-body dynamic and static PET/CT with low-dose [18F]PSMA-1007 in prostate cancer patients
Source: Eur J Nucl Med Mol Imaging. 2024 Jan 30;51(7):2137–50. doi: 10.1007/s00259-024-06620-1 (PMC11139746; doi:10.1007/s00259-024-06620-1)
Supplement: Supplementary file 1 — Supplementary file1 (DOCX 14 KB) [file 259_2024_6620_MOESM1_ESM.docx]

**Supplementary Table 1** Quantitative parameters of the background (spleen) and tumor lesions in terms of the objective evaluation of image quality of static PET/CT performed 70 min after tracer administration. Data are presented as mean ± standard deviation (mean ± SD).

| **Measurement** | **PET-10** | **PET-8** | **PET-6** | **PET-5** | **PET-4** | **PET-2** |
| --- | --- | --- | --- | --- | --- | --- |
| Tumor lesions SUV_mean_ | 7.80 ± 6.35 | 7.78 ± 6.30* | 7.71 ± 6.19* | 7.85 ± 6.30 | 7.86 ± 6.19 | 7.57 ± 6.07* |
| Tumor lesions SUV_max_ | 16.07 ± 17.44 | 16.12 ± 17.35 | 16.16 ± 17.11 | 16.39 ± 17.22 | 16.39 ± 17.45 | 16.83 ± 18.56 |
| TBR | 1.07 ± 0.86 | 1.07 ± 0.86 | 1.07 ± 0.86 | 1.09 ± 0.92 | 1.07 ± 0.86 | 1.08 ± 0.87 |
| Spleen SNR | 8.19 ± 3.04 | 7.75 ± 2.70* | 7.21 ± 2.34* | 6.90 ± 2.24* | 6.36 ± 2.13* | 5.48 ± 1.59* |

*SNR,* signal-to-noise ratio; *TBR,* tumor-to-background ratio.

SNR of the background was measured as spleen SUV_mean_ divided by spleen SD

TBR was defined as tumor lesion SUV_mean_ divided by spleen SUV_mean_

*Significant difference in comparison with the respective values of PET-10, p < 0.05
